# Contemplations on MDMX (MDM4) driving triple negative breast cancer circulating tumor cells and metastasis

**DOI:** 10.18632/oncotarget.27134

**Published:** 2019-08-20

**Authors:** Chong Gao, Gu Xiao, Jill Bargonetti

**Affiliations:** ^1^ The Department of Biological Sciences at Hunter College, Belfer Building, City University of New York, New York, NY, USA; ^2^ The Graduate Center Biology Program of City University of New York, New York, NY, USA; ^3^ Department of Cell and Developmental Biology, Weill Cornell Medical College New York, New York, NY, USA

**Keywords:** MDMX, MDM4, mutant p53, mtp53, metastasis

## Abstract

MDMX (MDM4) is emerging as an important breast cancer (BC) biomarker, and oncoprotein, that can be targeted in combination with its well-known family member MDM2. While MDM2 has previously been implicated in driving BC metastasis, information about the role of MDMX in driving circulating tumor cells (CTCs) and BC metastasis is lacking. BCs often have alterations of MDM2, MDMX, and mutant p53 (mtp53). Therefore, the role of MDM2 and MDMX in the context of mtp53 in BCs requires further clarification. Our group has recently reported that triple negative breast cancer (TNBC) metastasis is dependent on both MDM2 and MDMX, and depleting MDM2 results in increased MDMX, but depleting MDMX does not cause an increase in MDM2. In the context of human TNBC expressing mtp53 in an orthotopic mouse model the down-regulation of MDMX virtually cleared CTCs from the blood. Contemplations, using the available literature, suggest that disrupting the stability and/or function of MDMX protein (and its downstream targets), in the context of mtp53 expressing BCs, might be beneficial for patient survival. It remains to be determined if blocking mtp53-MDMX pathways can inhibit early stage TNBC and eliminate CTCs that have the potential to form metastatic lesions.

## INTRODUCTION

MDMX (MDM4) directly promotes tumor formation and promotes genomic instability [1, 2]. A number of manuscripts have suggested that blocking both MDMX and MDM2 is an ideal goal for cancer treatment [3–6]. Triple negative breast cancer (TNBC) is a subtype of BC that is characterized by transformed cells that lack expression of estrogen receptor (ER), progesterone receptor (PR) and human epidermal growth factor receptor 2 (HER2). TNBC is unable to be treated by well-identified hormone receptor pathway inhibitors. The cancer genome atlas network identified the tumor suppressor p53 pathway, including its MDM protein regulators, as one of three major pathways altered across all subtypes of BC (with 80% of TNBC harboring mutations in the *Tp53* gene) [7]. A detailed analysis focused on mtp53, MDM2 and MDMX expression in BCs in the METABRIC data set highlights the fact that up-regulation of these biomarkers sometimes co-occur in aggressive disease [8]. As MDMX gains traction as a biomarker that may drive metastasis and disease progression, its role in the context of mtp53 is proving to be important [3–5, 7–10]. When stratified with p53 mutational status, elevated MDMX expression and p53 mutations predict poor BC metastasis free survival [10]. Metastatic disease is one of the major causes for patient death. Therefore, determining the role played by MDMX in BC metastasis may hold the key to utilizing this biomarker for developing new treatments to reduce mortality for classes of BC currently lacking diagnostic and therapeutic options [10]. MDM2 and MDMX both block wild-type p53 tumor suppressor activity in mouse models [11]. However, while MDM2 functions as the key E3 ligase for wild-type p53, MDMX lacks intrinsic E3 ligase activity. The hetero-dimerization of MDM2 with MDMX stabilizes MDM2 and enhances its E3 ligase activity, resulting in efficient inhibition of wild-type p53 as well as proteasomal degradation of MDMX [12]. MDM2 promotes BC circulating tumor cells (CTCs) and down-regulation of MDM2 in an orthotopic mouse model shows reduced CTCs and significantly increases levels of E-cadherin [13]. MDMX increases cell proliferation in the context of TNBC cells that express mtp53 [5]. Our recent 2019 finding in Breast Cancer Research reports that MDMX promotes human TNBC MDA-MB-231 CTCs, and lung metastases, while correlating with up-regulation of the metastasis promoting G-protein coupled receptor C-X-C chemokine receptor type 4 (CXCR4) [9]. This information helps to explain the role played by MDMX in driving aggressive TNBC metastasis.

### MDMX and MDM2 promote TNBC circulating tumor cells

**Figure 1:**
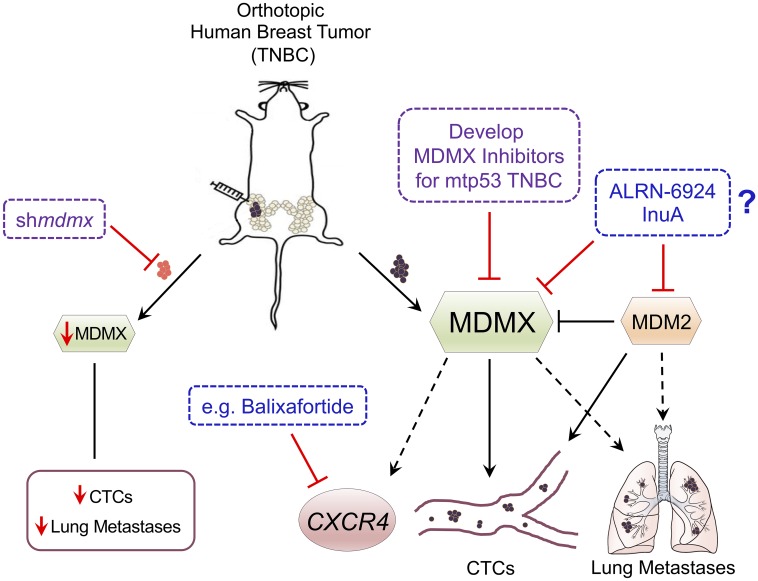
Schematic of MDMX promoting TNBC metastasis and proposed targeting strategies. The orthotopic immunodeficient NOD scid gamma (NSG) mouse model with human triple negative breast cancer (TNBC) contains either cells with endogenously elevated MDMX expression or cells engineered with MDMX knockdown (tumor indicated in red). Knockdown of MDMX blocks the presence of circulating tumor cells (CTCs) in the context of mtp53 expressing tumors and reduces metastasis to the lung. MDMX correlates with up-regulation of the metastasis promoting G-protein coupled receptor C-X-C chemokine receptor type 4 (CXCR4) *in vivo*. Some important questions remaining are if the development of new MDMX inhibitors, or if the existing inhibitors InuA or ALRN-6924 with or without the CXCR4 inhibitor Balixafortide can effectively inhibit TNBC progression.

We found that TNBC metastasis depends on both MDM2 and MDMX, and depleting MDM2 in this context results in increased MDMX, while depleting MDMX does not cause an increase in MDM2 [9]. This is because MDM2 is an E3 ubiquitin ligase for MDMX and therefore down-regulates the levels of MDMX protein [12]. Importantly, we demonstrated (using an orthotopic immunodeficient NOD scid gamma (NSG) mouse model) that knockdown of MDMX almost completely abolishes the presence of CTCs in the context of mtp53 expressing tumors and reduces metastasis to the lung [9] (see Figure 1). This data provides the first experimental proof of the metastatic-promoting activity of MDMX. Furthermore, the up-regulation of MDMX when MDM2 is inhibited highlights the importance of developing dual inhibitors for MDM2 and MDMX. Both MDM2 and MDMX have oncogenic power in the context of mtp53 [8]. Interestingly, MDMX, or MDM2, knockdown does not correlate with a less aggressive tumor local-stroma invasion pattern, suggesting that MDM proteins facilitate intravasation and/or TNBC survival in the blood stream [9]. In addition to regulating E-cadherin, MDM2 promotes immature vessel formation and increases pro-angiogenic vascular endothelial growth factor (VEGF) expression, providing potential mechanisms by which MDM2 drives metastasis [13]. It remains unknown whether MDMX similarly can regulate VEGF or E-cadherin, but we observed that MDMX expression positively correlates with increased tumor-associated CXCR4 expression [9]. Our data shows this relationship between MDMX and CXCR4 only occurs *in vivo* (see Figure 1)*,* thereby suggesting signaling events regulated by a tumor stroma interaction.

### The promise of small molecule inhibitors targeting both MDM2 and MDMX

The current state of the field suggests that disrupting the stability and/or function of MDM protein family members, in the context of mtp53 expressing BCs, might be beneficial for targeting early stage TNBC and eliminating CTCs that can go on to form metastatic lesions [3, 9, 13]. Furthermore, elucidating the underlying mechanisms by which these proteins contribute to BC metastasis could provide novel therapeutic windows for preventing and/or treating early metastasis in MDM2/MDMX-overexpressing TNBC patients. Importantly, the strong metastatic-promoting activity of MDMX uncovered by our group accentuates MDMX as a promising biomarker and therapeutic target. This suggests that it is important to develop MDMX antagonists for triple negative breast tumors harboring mtp53. The majority of therapeutic interventions targeting MDM2 and/or MDMX are designed to inhibit the interactions between MDM family members and p53, thus stabilizing wild-type p53 to activate cell cycle arrest or apoptosis [14]. The stapled peptide ALRN-6924, in clinical trials for hematological cancers and select solid tumors that retain wild-type p53, has not yet been tested in a TNBC model with mtp53 [3, 14]. An important question to be answered is whether ALRN-6924 can effectively inhibit TNBC progression (see Figure 1). Given the important roles that MDM2 and MDMX play in the context of mtp53 expressing cancers, strategies to inhibit the MDM2-MDMX interaction and degrade both proteins are becoming increasingly attractive. Notably, a natural inhibitor Inulanolide A (InuA) has been shown to suppress prostate cancer cell viability, migration and invasion via disrupting the MDM2-MDMX interaction by binding to the RING domains of these two proteins and leads to their degradation [15]. Importantly the anti-tumor/ anti-metastasis activity persists independent of p53, or androgen receptor, status [15]. We identified CXCR4 as an indirect target of MDMX in the context of tumor stroma communication. One potent CXCR4 blocker, Balixafortide, is the only CXCR4 inhibitor currently undergoing a clinical trial for patients with metastatic BCs and has given promising results for blocking metastasis [16]. A logical next step is to test whether combinatorial targeting of CXCR4 and MDMX might eliminate TNBC metastatic spread. Our findings, alongside the findings from other laboratories studying metastatic breast cancer, provide a model (Figure 1) for how down-regulating MDMX (and/or its downstream targets) might reduce CTCs and metastasis.
